# Decoding ascitic immunological niches with multi-modal machine learning reveals prognostic and chemoresistant determinants in ovarian cancer

**DOI:** 10.3389/fimmu.2025.1698793

**Published:** 2025-12-03

**Authors:** Lin Yang, Tianhui He, Jing Wang, Xiaolan Zhang, Lin Zeng, Qinkun Sun, Yuelin Song, Yufei Nie, Xinran Gao, Chunliang Shang, Hongyan Guo

**Affiliations:** 1 Department of Obstetrics and Gynecology, Peking University Third Hospital, Beijing, China; 2 Mass General Cancer Center, Massachusetts General Hospital, Harvard Medical School, MA, United States

**Keywords:** epithelial ovarian cancer, survival analysis, immunological niches, deep learning, platinum-based drug chemotherapy resistance prediction

## Abstract

**Background:**

Malignant ascites in high-grade serous ovarian cancer (HGSOC) represent a fluid extension of the tumor microenvironment, embedding immune programs that may inform prognosis and treatment response. We investigated whether ascitic T-cell phenotypes, integrated with clinical variables, improve prediction of overall survival (OS), progression-free survival (PFS), progression-free interval (PFI), and platinum-based drug chemotherapy resistance (P-DCR).

**Methods:**

We retrospectively analyzed 87 patients with FIGO III/IV HGSOC with treatment-naïve ascites treated at Peking University Third Hospital (May 2019–Mar 2024; median follow-up, 33 months). Ascites (>1,000 mL) underwent standardized processing and multiparametric flow cytometry to quantify T-cell subsets. To prevent information leakage, we used repeated nested cross-validation with event-stratified folds: inner folds performed endpoint-specific screening with Benjamini–Hochberg FDR control, redundancy reduction, and multicollinearity checks; clinical covariates were added by incremental contribution testing. Cox proportional hazards, Random Survival Forests (RSFs), and DeepSurv modeled survival endpoints; a random-forest classifier modeled P-DCR. Performance was summarized on outer folds [C-index for survival; receiver operating characteristic–area under the curve (ROC-AUC) for P-DCR]. Model interpretability used Shapley Additive Explanations (SHAP).

**Results:**

Across endpoints, combined clinical + ascites features outperformed single-source features, with RSF consistently best. Outer-fold testing C-indices for RSF with combined features were 0.72 (OS), 0.70 (PFS), and 0.74 (PFI). The P-DCR classifier achieved a mean AUC of 0.69 with combined features (accuracy, 0.66; sensitivity, 0.70; specificity, 0.62). Feature-count sensitivity analyses showed performance gains plateauing at modest *k* (≈5–7). Kaplan–Meier curves derived from combined-feature risk scores demonstrated clear stratification. SHAP analyses indicated protective effects of poly(ADP-ribose) polymerase (PARP) inhibitor maintenance across endpoints, while ascitic T-cell subsets, including PD-1^+^CD57^+^CD4^+^ and CCR7^-^CD45RA^+^CD4^+^ populations, were repeatedly associated with higher risk; age contributed strongly to PFI.

**Conclusions:**

Integrating ascitic immunophenotyping with clinical factors improves risk prediction in HGSOC, with RSF offering robust performance under rigorous, leakage-safe validation. Ascites-resident T-cell states provide complementary, reproducible prognostic signals for survival and platinum response, supporting their potential utility for patient stratification and hypothesis generation for immunomodulatory strategies.

## Introduction

Epithelial ovarian cancer (EOC) is recognized as the most lethal gynecologic malignancy due to its frequent late-stage diagnosis (stages III/IV), abundant ascitic fluid, and extensive pelvic and abdominal metastases (1, 2). Cytoreductive surgery combined with chemotherapy remains the first-line treatment (3, 4). However, most patients face poor prognosis due to chemotherapy resistance. This underscores an urgent need to explore the composition of the tumor microenvironment (TME) to identify new strategies for immunotherapy (5). We all agree that postoperative survival and recurrence are closely associated with the diameter of residual tumor after surgery; the larger the postoperative residual lesion, the shorter the postoperative recurrence interval and the shorter the overall survival (OS) (27, 28). Ascitic fluid is considered a diluted form of the TME (6), containing tumor cell clusters, immune cells, and soluble cytokines. Solid tumors establish dynamic immunological niches shaped by local immune reprogramming, metabolic stress, and stromal remodeling. In ovarian cancer, ascitic fluid provides a unique window into this niche, reflecting immune subset shifts and their functional states. While tumor-infiltrating lymphocytes have been extensively studied, the niche-specific relevance of ascitic immune cells in shaping prognosis and therapy resistance remains poorly characterized (7). Previous studies have shown that the phenotype and frequency of T cells in ovarian cancer ascitic fluid fall between those in tumors and peripheral blood, reflecting the immune state of the TME (8–10). The immune characteristics of ascites also influence patients’ disease status, tumor size, and postoperative residual tumor status (R0/R1/R2 classification). Recent studies have demonstrated that immune cell subsets, including CD8^+^ T cells and Programmed Death 1 (PD-1)^+^ T cells, are closely associated with tumor progression and resistance to chemotherapy in patients with high-grade serous ovarian cancer (HGSOC). Zhang et al. (2023) (26) showed that high levels of CD8^+^ T-cell infiltration in ascitic fluid correlated with better survival outcomes in patients with HGSOC, while elevated PD-1^+^ T cells were linked to chemotherapy resistance. Therefore, investigating the impact of T-cell subsets in the tumor and ascitic fluid of patients with HGSOC on prognosis is of significant value.

The Cox proportional hazards (CPH) model is a classic method for survival analysis. However, its linear and proportional hazard assumptions limit its ability to handle complex, nonlinear, and dynamic relationships (11), especially in multimodal, high-dimensional data (12–15). In recent years, deep learning methods and machine learning models [such as Random Survival Forests (RSFs)] have provided new solutions for survival analysis. Through neural network architectures, deep survival models can capture complex nonlinear relationships, integrate multimodal data, and improve predictive performance (16–18). Furthermore, machine learning methods have been widely applied in related studies, enabling the extraction of latent patterns and rules in data and the effective selection and modeling of various features (19).

Moreover, with the incorporation of interpretability tools (20) such as Shapley Additive Explanations (SHAP), deep learning can not only reveal the impact of key features but also enhance the clinical applicability of the models. RSF, in particular, naturally inherits interpretability from its tree-based structure, allowing researchers to assess how individual features (e.g., ascitic immune cell characteristics) influence survival outcomes and treatment resistance through aggregated tree predictions.

In this study, we developed a deep survival model for predicting OS and progression-free survival (PFS) while utilizing a machine learning model to evaluate platinum-based drug chemotherapy resistance (P-DCR). By incorporating interpretability tools such as SHAP (21–23), we analyzed the associations between ascitic immune cell characteristics and HGSOC patient survival and P-DCR. This provides theoretical support and practical guidance for optimizing individualized treatment strategies.

## Methods

### Data source

This study included patients with HGSOC at FIGO stage III/IV with ascites. These patients were newly diagnosed and completed first-line treatment at Peking University Third Hospital from May 2019 to March 2024. Patients with other ovarian diseases, infectious/blood/kidney/liver diseases, other tumors, prior anti-tumor treatments (surgery, chemotherapy, radiotherapy, and immunotherapy) within 5 years, or lost to follow-up were excluded. Patients who had received anti-tumor treatments such as surgery, chemotherapy, radiotherapy, and immunotherapy within 5 years before enrollment, as well as those who were lost to follow-up during the follow-up process, were also excluded. Finally, 87 patients met the criteria and were enrolled. The basic characteristics of all patients with HGSOC were collected, including age, stage, lymph node metastasis (LNM), surgical method, surgical satisfaction, treatment regimen, treatment efficacy, chemotherapy sensitivity, and maintenance treatment. OS refers to the time from diagnosis to death from any cause, with patients remaining alive at the end of follow-up censored; PFS is the time from diagnosis to the first occurrence of disease progression or death, with patients having no such events censored at the end of follow-up; and PFI (post-chemotherapy progression-free interval) denotes the time from the end of initial treatment to the occurrence of tumor progression. The results of resection surgery were defined as no grossly visible residual tumor (R0), residual tumor lesions ≤ 1 cm (R1), or residual tumor lesions > 1 cm (R2). R1 and R2 were collectively referred to as NR0. The best efficacy of first-line treatment, complete remission (CR), was defined as normal serum CA125 level, normal physical examination, and no signs of recurrence on computed tomography (CT) scan. Patients who did not achieve CR, including those with partial remission, stable disease, and progressive disease, were generally referred to as non-CR (NCR). PFI (in months) referred to the time from the end of first-line treatment to the determination of cancer progression (including CA125 elevation and imaging evidence) by clinicians. Patients with PFI < 6 months were called platinum-resistant to chemotherapy, and patients with PFI > 6 months were called platinum-sensitive to chemotherapy. PFS referred to the time from diagnosis to recurrence (in months). OS referred to the time from diagnosis to death (in months). The follow-up ended on March 2024. The median follow-up time was 33.00 (6.00–56.00) months.

### Sample collection, processing, and flow cytometry detection

To ensure flow cytometry accuracy, immune cell detection was only performed on ascites with a volume > 1,000 mL. Ascites samples were collected during the surgical procedure. All samples were processed within 1 h after collection. The ascites was centrifuged at 2,000 *g* for 10 min at 4°C. Density gradient centrifugation was performed using Ficoll (1.077, GE Healthcare, USA) and phosphate-buffered saline (PBS) in a ratio of 1:1.5 to obtain mononuclear cells. The cells were collected and washed twice with PBS, followed by centrifugation at 500 g for 5 min. Then, the cells were manually counted and temporarily stored at 0°C to complete staining as soon as possible. At room temperature, the cells (1 × 10^6^) were stained with specific monoclonal antibodies (mAbs) in the dark for 15 min. Then, the cells were fixed with 1% paraformaldehyde. Flow cytometry analysis was performed using a CytoFLEX S (Beckman Coulter). The data were analyzed using Cytoexpert v. 2.3 software.

### Data preprocessing

To prevent information leakage, feature screening was conducted exclusively within training folds of a nested cross-validation framework, with held-out folds used only for evaluation. Clinically plausible covariates were pre-specified and, where alternatives existed, adjudicated by incremental contribution testing within the inner folds; variables showing consistent improvement in discrimination were retained. Immune-cell candidates were screened per inner fold using the following: (i) endpoint-appropriate univariate tests—CPH for OS/PFS/PFI and Mann–Whitney *U* test for PDCR—with Benjamini–Hochberg false discovery rate (FDR) adjustment [reporting hazard ratios (HRs) with 95% confidence intervals (CIs)]; (ii) redundancy reduction by pairwise correlation; and (iii) multicollinearity assessment via variance inflation factors (VIFs). Correlation heatmaps and VIF summaries are provided in the Supplementary Materials.

### Feature group selection for survival outcomes

After the first-stage nested screening, an endpoint-specific immune feature subset was fixed as the baseline model. Clinical covariates were then evaluated by incremental contribution testing under the same nested cross-validation framework: within inner folds, each clinical variable was added one at a time to the baseline immune set and then cumulatively in a pre-specified order, with models refit at each step and performance compared on held-out data. A covariate was retained if it produced a consistent improvement in discrimination (C-index) across inner folds; the minimal clinical augmentation achieving the highest mean performance defined the final feature group for that endpoint. Outer-fold results were summarized as mean C-index.

### Construction and training of the deep survival model and random forest P-DCR prediction model

**Figure 1:**
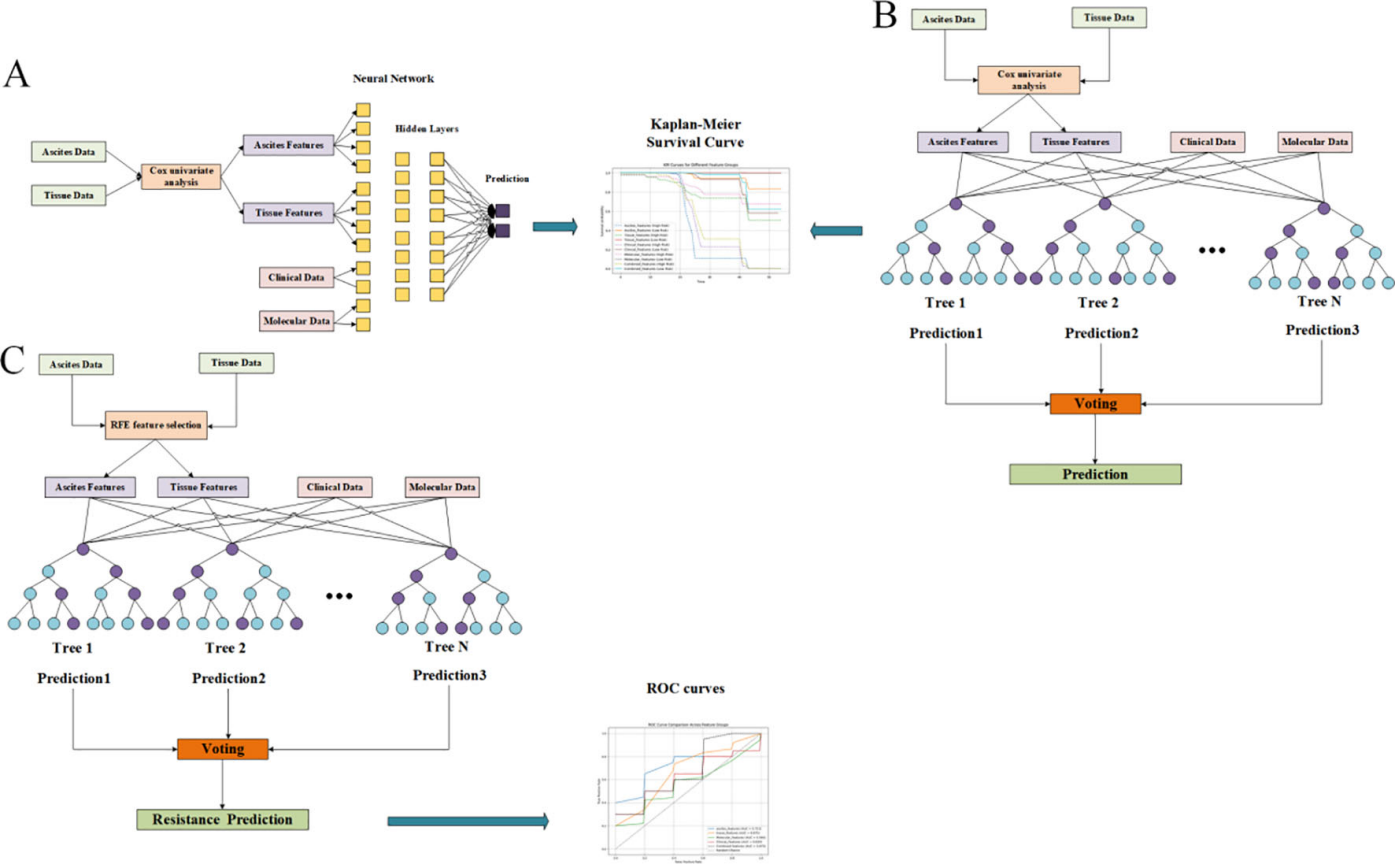
Feature screening diagrams (Display mode 1). **(A)** Overall survival (OS) related to ascites indicators. **(B)** Progression-free survival (PFS) of ascites indicators. **(C)** Progression-free interval (PFI) related to ascites indicators.

CPH, RSF, and DeepSurv were evaluated for survival outcomes, and a random-forest classifier was used for PDCR. All models were trained under a repeated nested cross-validation scheme with stratified sampling based on survival outcomes (event vs. non-event) to mitigate overfitting in a small, high-dimensional setting and balance the event ratio across folds. Feature screening and hyperparameter tuning were confined to inner folds, and outer folds were reserved for evaluation. The CPH model was fitted by ridge-penalized partial likelihood (λ = 0.10) under proportional-hazards assumptions, with multicollinearity addressed during inner-fold screening. The RSF configuration (**Figure 1B**) employed log-rank splitting with 200 trees, max_depth = 8, and min_samples_leaf = 5 after inner-loop optimization. The DeepSurv architecture (**Figure 1A**) consisted of a compact multilayer perceptron with two hidden layers (64, 32; ReLU) producing a single linear log-risk output and trained by the negative Cox partial likelihood (i.e., not direct survival-time prediction); optimization used Adam with Reduce-on-Plateau scheduling (initial learning rate 1 × 10^-^³), dropout = 0.20, weight decay = 1 × 10^-4^, batch size = 16, and early stopping based on inner-fold validation loss. For PDCR, a random-forest classifier (**Figure 1C**) with 300 trees, max_depth = 8, min_samples_leaf = 3, and class_weight = “balanced” was adopted to address class imbalance. Performance across outer folds is summarized as mean ± SD of C-index (survival) and area under the curve (AUC) (PDCR) with bootstrap 95% CIs; precision–recall curves, confusion matrices, and paired between-model comparisons are reported in Results. To characterize model behavior and potential overfitting, training-versus-validation learning curves were recorded (partial-likelihood/C-index trajectories for CPH and DeepSurv; out-of-bag plus inner-fold validation curves for RSF and the PDCR classifier) and used to verify optimization convergence and quantify the train–validation generalization gap.

### Interpretability analysis

Continuous variables were summarized as mean ± SD; 95% CIs were obtained via percentile bootstrap. Distributional assumptions were assessed with the Shapiro–Wilk test. Two-group comparisons used the Wilcoxon rank-sum test. For survival endpoints, univariate associations were estimated with CPH models (robust variance), reporting HRs with 95% CIs; multiplicity across immune markers was controlled using the Benjamini–Hochberg FDR, with adjusted *q*-values tabulated. Redundancy and multicollinearity were examined using Spearman pairwise correlation matrices/heatmaps and VIFs, with details provided in the Supplementary Materials. Predictive performance was estimated using repeated nested cross-validation with event-stratified folds to preclude information leakage; survival models were evaluated by Harrell’s concordance index and classification models by ROC AUC and average precision. Outer-fold metrics are presented as mean ± SD with bootstrap 95% CIs.

## Results

We enrolled 87 patients, of whom 45 (51.72%) were in stage III and 42 (48.28%) were in stage IV. The average preoperative serum CA125 of the above patients was 1,136 U/mL. The median age of the patients was 56 years. A total of 41 patients received primary debulking surgery (PDS), and 46 patients received ascites biopsy/biopsy surgery and two to four cycles of NACT, followed by IDS. R0 was achieved in 48 patients, and NR0 was achieved in 39 patients. All patients received chemotherapy after debulking surgery, and CR was achieved in 78 patients (89.66%). After first-line treatment, 51 (58.62%) patients were treated with poly(ADP-ribose polymerase) inhibitors (PARPi). By the end of the follow-up, 46 (52.87%) patients had relapsed, of which 17 were chemotherapy-resistant relapses and 29 were chemotherapy-sensitive relapses. A total of 23 patients died after relapse. The clinical characteristics of the included patients are summarized in **Table 1**.

**Table 1:** Characteristics of the enrolled patients.

Characteristics	Ascites (*n* = 87)
Age (years)	55.94 ± 9.71
FIGO stage	
Phase III	45 (51.72%)
Phase IV	42 (48.28%)
Serum CA125 (U/mL)	1136 (585.05, 2,310.00)
Surgical procedure	
PDS	41 (47.13%)
NACT+IDS	46 (52.87%)
Surgical satisfaction	
R0	48 (55.17%)
NR0	39 (44.83%)
Treatment efficacy	
CR	78 (89.66%)
NCR	9 (10.34%)
PARPi maintenance treatment	
PARPi	51 (58.62%)
None	36 (41.38%)
Chemosensitivity	
Sensitive	66 (75.86%)
Resistant	17 (19.54%)
Under assessment	4 (4.60%)
Vital status at last follow-up	
Alive	64 (73.56%)
Deceased	23 (26.44%)
Relapse	46 (52.87%)
Non-relapse	41 (47.13%)

### Statistical analysis results of ascites characteristics

**Figure 2:**
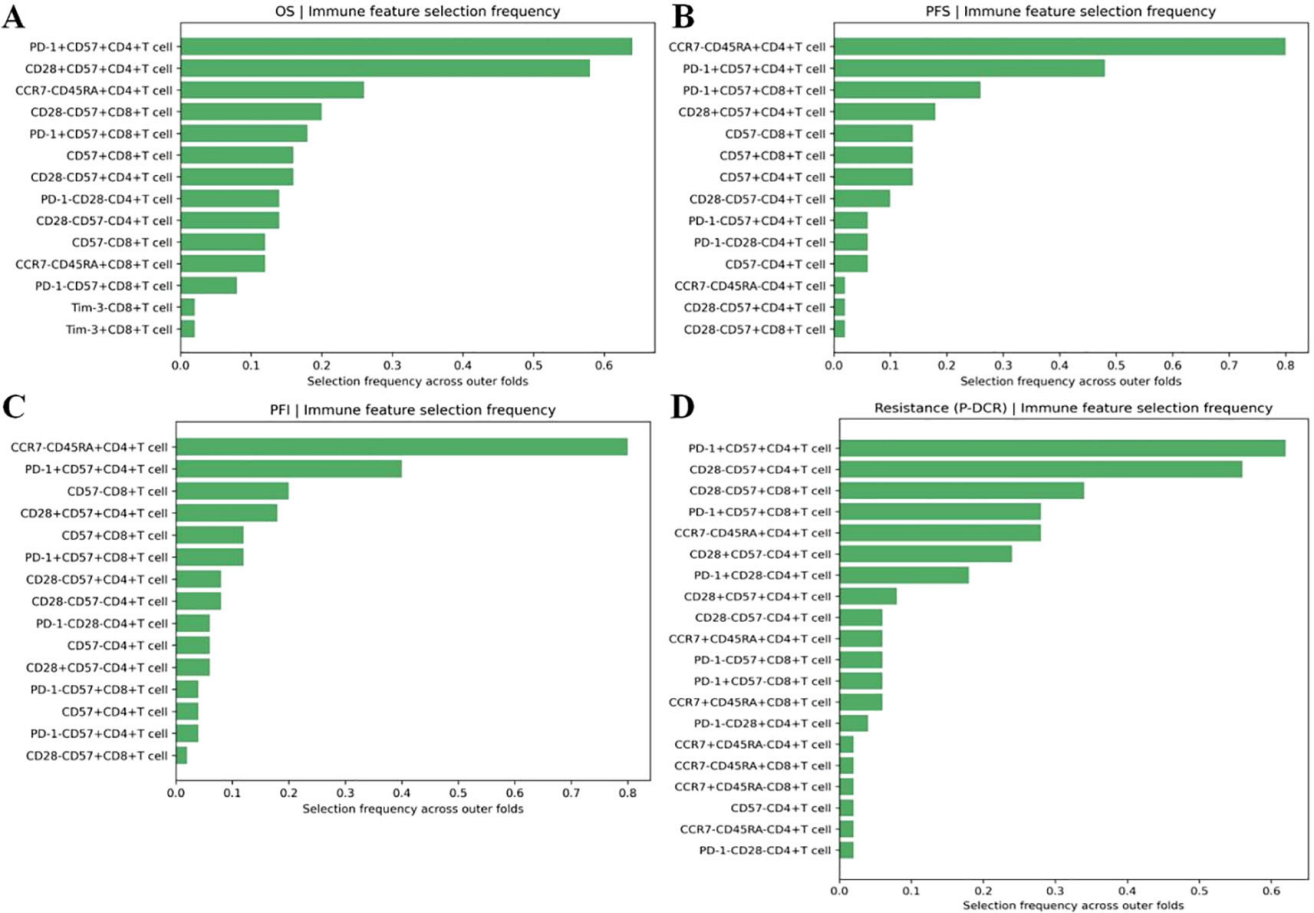
**(A–D)** Selection frequency of ascites immune features across outer folds: selection frequency bar plots for immune features associated with **A**: OS, **B**: PFS, **C**: PFI, and **D**: PDCR.

**Figure 3:**
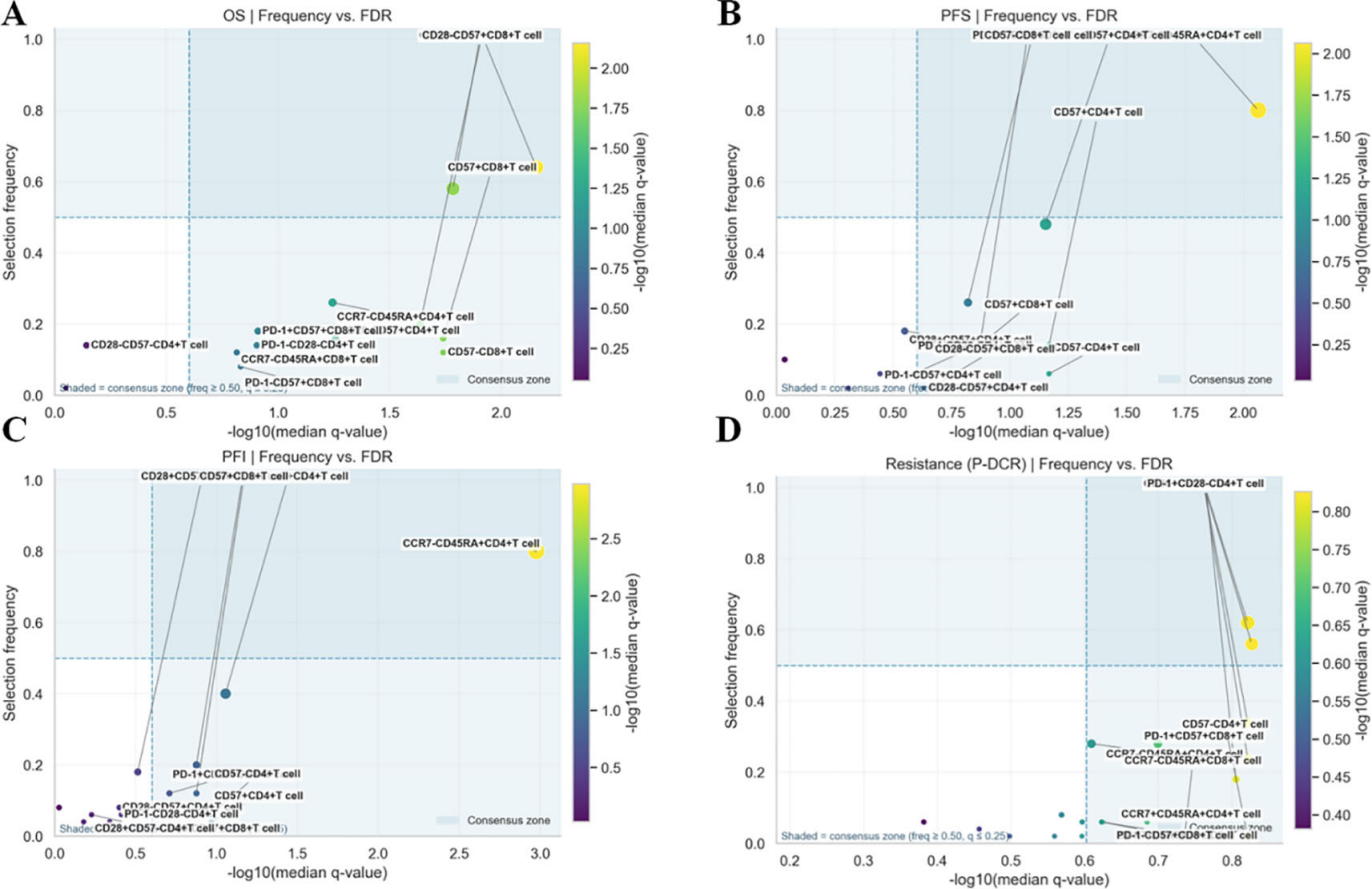
**(A–D)** Evidence–stability plots for ascites immune features: frequency vs. −log10(median q) scatter plots for each endpoint (**A**: OS, **B**: PFS, **C**: PFI, and **D**: PDCR).

**Figure 4:**
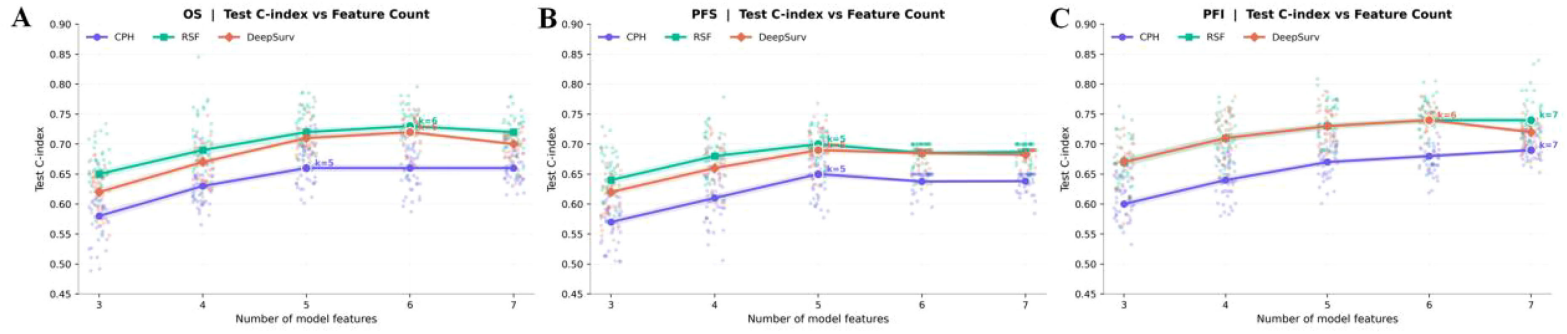
**(A–C)** Feature count sensitivity for survival outcomes (**A**: OS, **B**: PFS, **C**: PFI). Lines show test C-index means for CPH, RSF, and DeepSurv across *k* = 3–7.

Within the nested, within-fold screening framework, several ascites-derived immune phenotypes showed reproducible associations with survival endpoints after Benjamini–Hochberg FDR control. Screening stability across outer folds is summarized by selection frequency (**Figures 2A–D**; A = OS, B = PFS, C = PFI, and D = PDCR), and evidence–stability concordance is visualized as selection frequency vs. −log10(median *q*) (**Figures 3A–D**), with dashed lines denoting the *a priori* consensus thresholds (frequency ≥ 0.50; median *q* ≤ 0.25). Features in the upper-right quadrant and explicitly labeled in **Figure 4** constitute the consensus set carried forward to multivariable modeling.

Consistent patterns emerged across endpoints and are enumerated in **Table 2**. For OS, PD-1^+^CD57^+^CD4^+^ T cells were stably retained (frequency = 0.64) with a univariate Cox estimate HR = 2.12 (95% CI, 1.34–3.36); CD28^+^CD57^+^CD4^+^ T cells also showed stability (0.58) with HR = 1.83 (1.16–2.88), while CD28^-^CD57^+^CD8^+^ T cells appeared less frequently [0.20; HR = 1.61 (1.05–2.46)]. For PFS and PFI, CCR7^-^CD45RA^+^CD4^+^ T cells led the rankings (both frequency = 0.80) with HR = 1.96 (1.28–3.00) for PFS and HR = 2.24 (1.50–3.34) for PFI; PD-1^+^CD57^+^CD4^+^ T cells showed intermediate stability (PFS, 0.48; PFI, 0.40) with effect sizes consistent with risk increase (**Table 2**). For the PDCR endpoint (platinum resistance vs. sensitivity), discriminatory phenotypes are summarized by selection frequency and median *q* (no HR is reported because PDCR is binary); PD-1^+^CD57^+^CD4^+^ T cells (0.62), CD28^-^CD57^+^CD4^+^ T cells (0.56), and CD28^-^CD57^+^CD8^+^ T cells (0.34) ranked highest (**Figure 2D**, **Table 2**). Diagnostic assessments addressing redundancy and multicollinearity are provided in the Supplementary Materials (**Supplementary Figures S1**–**S3**).

**Table 2:** Ascites immune phenotypes prioritized by nested, within-fold screening.

Endpoint	Feature	Frequency	Median *q*	Score	HR	HR_CI_low	HR_CI_high
OS	PD-1^+^CD57^+^CD4^+^ T cell	0.64	0.007	0.748	2.12	1.34	3.36
	CD28^+^CD57^+^CD4^+^ T cell	0.58	0.016	0.653	1.83	1.16	2.88
	CD28^−^CD57^+^CD8^+^ T cell	0.20	0.023	0.366	1.61	1.05	2.46
PFS	CCR7^−^CD45RA^+^CD4^+^ T cell	0.80	0.009	0.860	1.96	1.28	3
	PD-1^+^CD57^+^CD4^+^ T cell	0.48	0.070	0.501	1.44	0.98	2.12
	PD-1^+^CD57^+^CD8^+^ T cell	0.26	0.151	0.298	1.31	0.9	1.9
PFI	CCR7^−^CD45RA^+^CD4^+^ T cell	0.80	0.001	0.860	2.24	1.5	3.34
	PD-1^+^CD57^+^CD4^+^ T cell	0.40	0.088	0.385	1.37	0.95	1.98
	CD57^−^CD8^+^ T cell	0.20	0.133	0.226	1.26	0.87	1.82
PDCR	PD-1^+^CD57^+^CD4^+^ T cell	0.62	0.151	0.730			
	CD28^−^CD57^+^CD4^+^ T cell	0.56	0.149	0.692			
	CD28^−^CD57^+^CD8^+^ T cell	0.34	0.151	0.534			

### Results of the feature group selection for survival outcomes

Shown in **Figure 4**; **Table 3**, across OS, PFS, and PFI, test C-index rose from smaller sets (*k* ≈3–4) and then plateaued; the optimal *k* varied by endpoint. For OS, RSF peaked at *k* = 6 (0.73), DeepSurv at *k* = 6 (0.72), and CPH at *k* = 5 (0.66). For PFI, RSF and DeepSurv co-peaked at *k* = 6 (0.74), while CPH peaked at *k* = 7 (0.69). For PFS, all models peaked at *k* = 5 (RSF 0.70; DeepSurv 0.69; CPH 0.65). Final clinical features: OS—age, stage, and PARPi; PFI—age, stage, serum CA125, and PARPi; PFS—stage and PARPi.

**Table 3:** Best *k* by model and endpoint with final clinical features.

Endpoint	Model	Best *k*	Best test mean	Final *k*	Final clinical features
OS	CPH	5	0.66	6	Age, stage, PARPi
	DeepSurv	6	0.72		
	RSF	6	0.73		
PFI	CPH	7	0.69	7	Age, stage, serum CA125, PARPi
	DeepSurv	6	0.74		
	RSF	6	0.74		
PFS	CPH	5	0.65	5	Stage, PARPi
	DeepSurv	5	0.69		
	RSF	5	0.70		

### Results of the survival models

After feature selection, three survival models—CPH, RSF, and DeepSurv—were established to predict OS, PFS, and PFI using three feature groups: Clinical_features (clinical-only), Ascites_features (ascites-only), and Combined_features (clinical-ascites combined); model performance was quantified by the concordance index, with detailed results in **Table 4**. For OS prediction, the Combined_features group outperformed the two single-feature groups across all models, and RSF exhibited the best performance: its training and testing C-indexes for Combined_features were 0.74 ± 0.04 and 0.72 ± 0.05, respectively, surpassing CPH (training: 0.68 ± 0.05; testing: 0.66 ± 0.06) and DeepSurv (training: 0.75 ± 0.03; testing: 0.70 ± 0.06), while in single-feature groups, RSF also led (Clinical_features: testing C-index 0.64 ± 0.10; Ascites_features: 0.69 ± 0.09) over CPH and DeepSurv. A similar trend was observed for PFS: the Combined_features group improved performance for all models, with RSF achieving the highest testing C-index (0.70 ± 0.04) in this group (training: 0.73 ± 0.04), outperforming CPH (testing: 0.65 ± 0.05) and DeepSurv (testing: 0.69 ± 0.06), and RSF also maintained advantages in single-feature groups (Clinical_features: testing 0.64 ± 0.11; Ascites_features: 0.67 ± 0.08). For PFI prediction, the Combined_features group remained superior, and RSF again delivered the highest testing C-index (0.74 ± 0.06) among all model-feature combinations (training: 0.76 ± 0.05), exceeding CPH (testing: 0.69 ± 0.06) and DeepSurv (testing: 0.72 ± 0.05); in single-feature groups, RSF’s testing C-indexes were 0.69 ± 0.09 (Clinical_features) and 0.72 ± 0.09 (Ascites_features), which were higher than those of CPH and DeepSurv. Collectively, the Combined_features group significantly enhanced predictive performance across all three endpoints compared to single-feature groups, and RSF consistently outperformed CPH and DeepSurv in the testing set. The training and validation loss curve are shown in the Supplementary Materials (**Supplementary Figure S4**).

**Figure 5:**
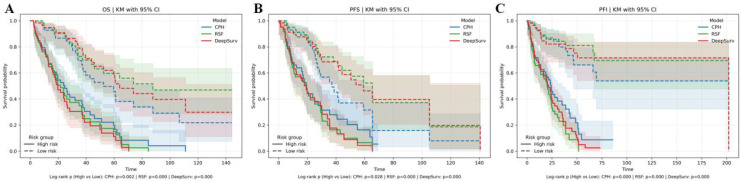
Kaplan–Meier (KM) curves for three models. **(A)** KM curves depicting overall survival (OS). **(B)** KM curves representing progression-free survival (PFS). **(C)** KM curves illustrating progression-free interval (PFI).

Kaplan–Meier curves (**Figure 5**) visually validated these findings, showing distinct survival stratification by risk groups derived from the combined feature models. Despite these improvements, DeepSurv consistently underperformed compared to RSF across all endpoints (**Table 4**), highlighting the latter’s superior ability to handle complex feature interactions.

**Table 4:** Performance comparison between models and feature groups.

Feature group	Model	Training C-index (mean ± SD)	Testing C-index (mean ± SD)
OS_Clinical	CPH	0.63 (± 0.08)	0.60 (± 0.08)
	RSF	0.70 (± 0.06)	0.64 (± 0.10)
	DeepSurv	0.74 (± 0.07)	0.62 (± 0.11)
OS_Ascites	CPH	0.67 (± 0.05)	0.64 (± 0.12)
	RSF	0.73 (± 0.09)	0.69 (± 0.09)
	DeepSurv	0.75 (± 0.09)	0.67 (± 0.10)
OS_Combined	CPH	0.68 (± 0.05)	0.66 (± 0.06)
	RSF	0.74 (± 0.04)	0.72 (± 0.05)
	DeepSurv	0.75 (± 0.03)	0.70 (± 0.06)
PFS_Clinical	CPH	0.64 (± 0.06)	0.61 (± 0.10)
	RSF	0.68 (± 0.05)	0.64 (± 0.11)
	DeepSurv	0.71 (± 0.05)	0.63 (± 0.12)
PFS_Ascites	CPH	0.66 (± 0.07)	0.63 (± 0.12)
	RSF	0.71 (± 0.06)	0.67 (± 0.08)
	DeepSurv	0.73 (± 0.09)	0.66 (± 0.09)
PFS_Combined	CPH	0.67 (± 0.04)	0.65 (± 0.05)
	RSF	0.73 (± 0.04)	0.70 (± 0.04)
	DeepSurv	0.74 (± 0.06)	0.69 (± 0.06)
PFI_Clinical	CPH	0.70 (± 0.09)	0.66 (± 0.09)
	RSF	0.74 (± 0.07)	0.69 (± 0.09)
	DeepSurv	0.76 (± 0.09)	0.68 (± 0.10)
PFI_Ascites	CPH	0.72 (± 0.09)	0.68 (± 0.13)
	RSF	0.76 (± 0.08)	0.72 (± 0.09)
	DeepSurv	0.78 (± 0.06)	0.70 (± 0.12)
PFI_Combined	CPH	0.71 (± 0.06)	0.69 (± 0.06)
	RSF	0.76 (± 0.05)	0.74 (± 0.06)
	DeepSurv	0.78 (± 0.05)	0.72 (± 0.05)

### P-DCR prediction results

**Figure 6:**
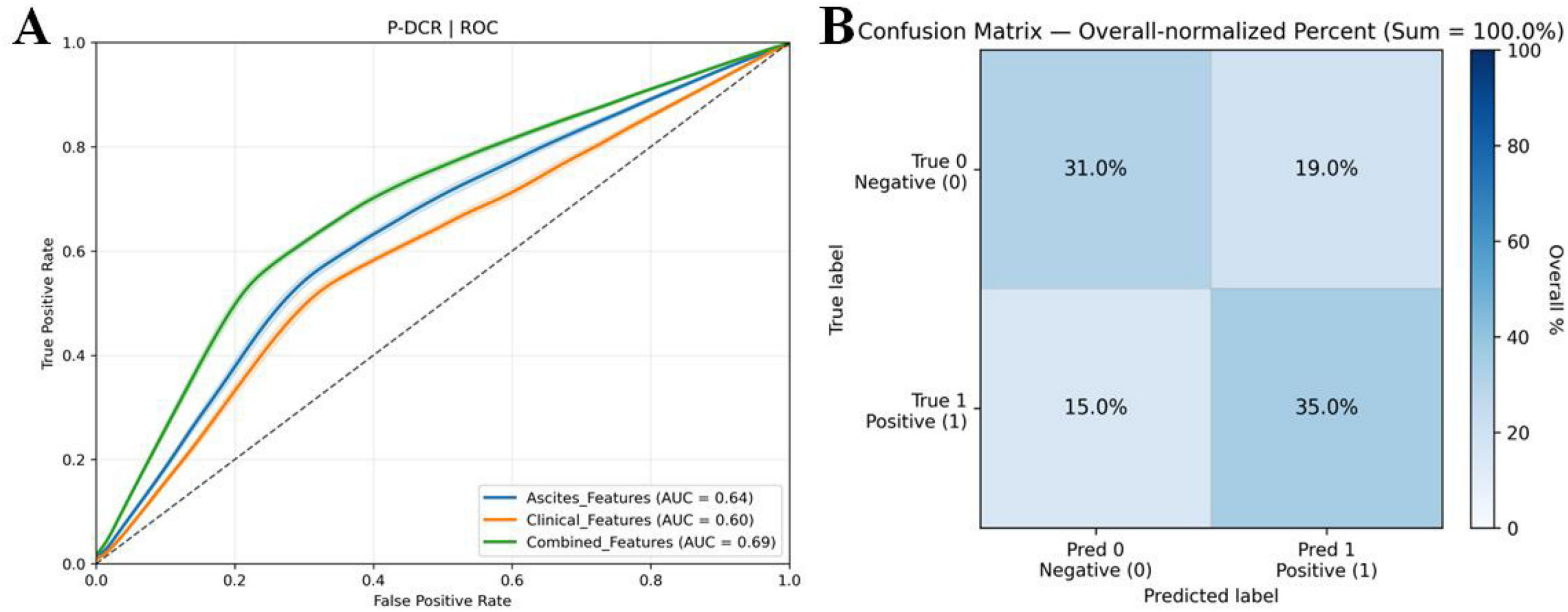
**(A)** ROC curves for comparison of multiple P-DCR prediction models. ROC curves illustrating the discriminative performance of diverse models in predicting P-DCR. **(B)** Confusion matrix derived from 3,000 bootstrap iterations, showing the trade-off between true positive rate and false positive rate for model evaluation.

In P-DCR prediction (**Table 5**), models were constructed based on the selected feature set. Classification performance metrics were compared using ROC curves (**Figure 6**). **Table 5** shows the performance metrics for different feature groups. The Ascites_features group had a mean AUC of 0.64 (± 0.05), a mean accuracy of 0.63 (± 0.07), a mean sensitivity of 0.54 (± 0.09), and a mean specificity of 0.71 (± 0.11). The Clinical_features group had a mean AUC of 0.60 (± 0.06), a mean accuracy of 0.62 (± 0.08), a mean sensitivity of 0.52 (± 0.10), and a mean specificity of 0.69 (± 0.12). The Combined_features group outperformed the others, with a mean AUC of 0.69 (± 0.04), a mean accuracy of 0.66 (± 0.09), a mean sensitivity of 0.70 (± 0.13), and a mean specificity of 0.62 (± 0.14). Training and validation loss are presented in the Supplementary Materials (**Supplementary Figure S5**).

**Table 5:** The performance for P-DCR prediction with different feature groups.

Feature group	AUC	Accuracy	Sensitivity	Specificity
Ascites_features	0.64 (± 0.05)	0.63 (± 0.07)	0.54 (± 0.09)	0.71 (± 0.11)
Clinical_features	0.60 (± 0.06)	0.62 (± 0.08)	0.52 (± 0.10)	0.69 (± 0.12)
Combined_features	0.69 (± 0.04)	0.66 (± 0.09)	0.70 (± 0.13)	0.62 (± 0.14)

### Feature interpretability results

**Figure 7:**
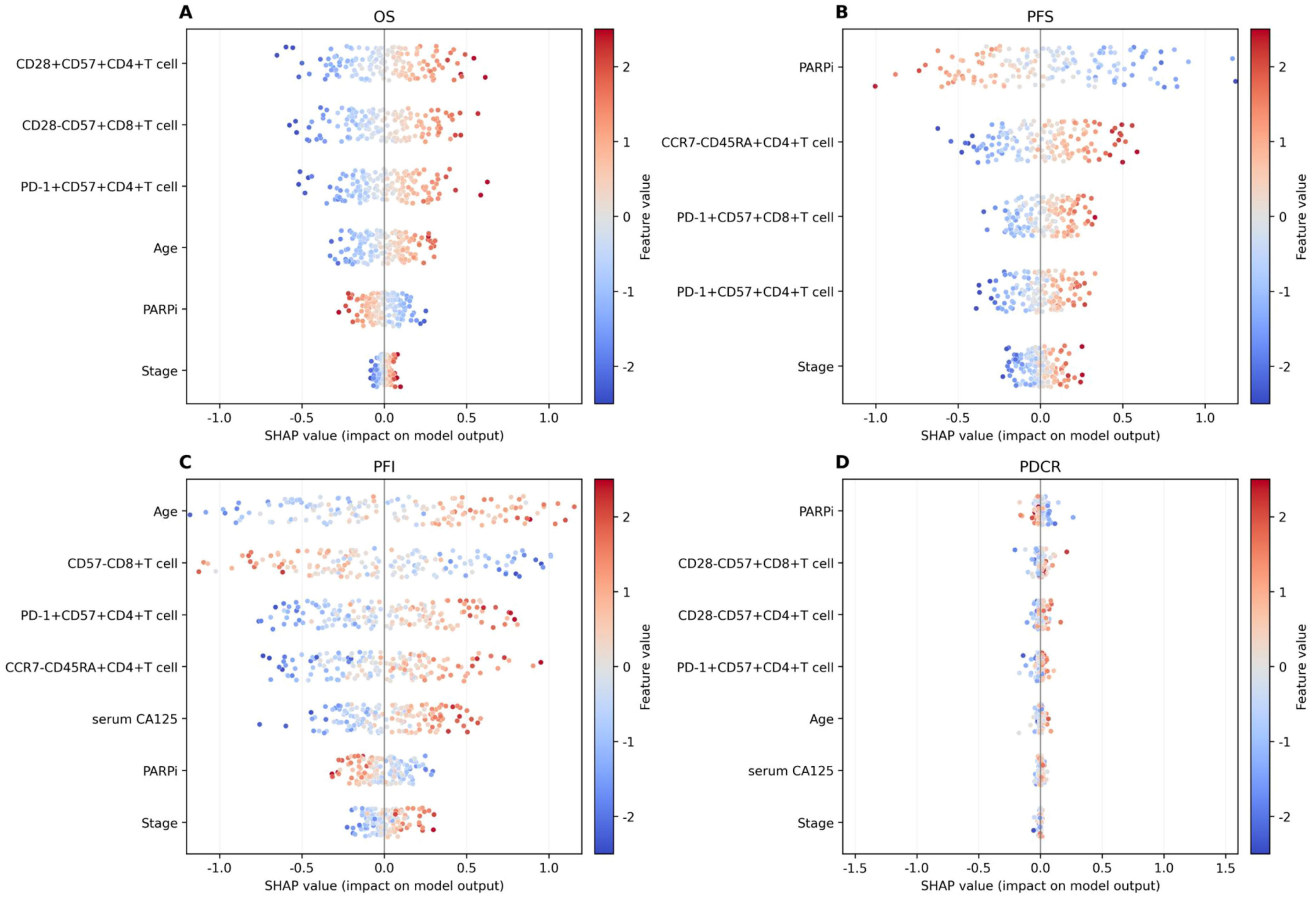
SHAP value for interpretability value visualization. **(A)** Overall survival (OS). **(B)** Progression-free survival (PFS). **(C)** Progression-free interval (PFI). **(D)** P-DCR.

**Figure 7**; **Tables 6**–**9** showcase the feature contributions and their respective importance rankings within diverse models, as determined by SHAP analysis. A positive SHAP value indicates a higher likelihood of poor prognosis and platinum resistance to chemotherapy, while a negative SHAP value indicates a better prognosis and platinum sensitivity to chemotherapy. The influence of key features on predictive outcomes was not uniform across all models.

**Table 6:** The SHAP value for OS prediction.

Feature	Total absolute SHAP value	Direction
CD28^+^CD57^+^CD4^+^ T cell	30.32	Positive
CD28^−^CD57^+^CD8^+^ T cell	29.4	Positive
PD-1^+^CD57^+^CD4^+^ T cell	25.54	Positive
Age	19.19	Positive
PARPi	13.17	Negative
Stage	4.08	Positive

**Table 7:** The SHAP value for PFS prediction.

Feature	Total absolute SHAP value	Direction
PARPi	52.79	Negative
CCR7^−^CD45RA^+^CD4^+^ T cell	32.56	Positive
PD-1^+^CD57^+^CD8^+^ T cell	20	Positive
PD-1^+^CD57^+^CD4^+^ T cell	18	Positive
Stage	12.68	Positive

**Table 8:** The SHAP value for PFI prediction.

Feature	Total absolute SHAP value	Direction
Age	82.42	Positive
CD57^−^CD8^+^ T cell	67.3	Negative
PD-1^+^CD57^+^CD4^+^ T cell	50.17	Positive
CCR7^−^CD45RA^+^CD4^+^ T cell	44.03	Positive
Serum CA125	34.04	Positive
PARPi	17.83	Negative
Stage	12.47	Positive

**Table 9:** The SHAP value for P-DCR prediction.

Feature	Total absolute SHAP value	Direction
PARPi	4.56	Negative
CD28^−^CD57^+^CD8^+^ T cell	3.84	Positive
CD28^−^CD57^+^CD4^+^ T cell	3.60	Positive
PD-1^+^CD57^+^CD4^+^ T cell	3.36	Positive
Age	2.28	Positive
Serum CA125	1.92	Positive
Stage	0.72	Positive

In the SHAP analysis of the deep survival model, the feature with the most significant impact on OS risk scores was CD28^+^CD57^+^CD4^+^ T cell, with the highest total absolute SHAP value (32.56) in **Table 6**. For PFS prediction, PARPi showed the most prominent influence, with the highest total absolute SHAP value (52.79) in **Table 7**. In PFI prediction, age emerged as the most impactful feature, with a total absolute SHAP value of 82.42 (**Table 8**). For platinum-based drug chemotherapy disease control rate (P-DCR) prediction, PARPi had the highest total absolute SHAP value (4.56) in **Table 9**.

Additionally, PARPi exerted a consistent negative effect on OS, PFS, PFI, and P-DCR, as indicated by negative direction values in **Tables 6**–**9**. Other notable features included CD28^−^CD57^+^CD8^+^ T cell (positive direction, total absolute SHAP value = 29.4) in OS prediction, CCR7^−^CD45RA^+^CD4^+^ T cell (positive direction, 32.56) in PFS prediction, CD57^−^CD8^+^ T cell (negative direction, 67.3) in PFI prediction, and CD28^−^CD57^+^CD8^+^ T cell (positive direction, 3.84) in P-DCR prediction.

## Discussion

Recent advances in spatial immunology and machine learning enable high-resolution decoding of immune microenvironments. By combining flow cytometric profiling of ascitic T-cell subsets with interpretable ensemble modeling, we aimed to explore the potential relationships between survival, recurrence, and P-DCR in patients with ovarian cancer and to construct deep learning models to improve the predictive performance of survival analysis. By integrating multi-omics and machine and deep learning methods, we sought to enhance the current prognosis evaluation for ovarian cancer, thereby supporting individualized treatment strategies.

In this study, a comprehensive comparative analysis was carried out to assess the influence of different feature sets on model performance in predicting OS, PFS, PFI, and P-DCR.

In survival predictions, the RSF model with combined clinical-ascites features outperformed single feature sets across all endpoints: OS (C-index = 0.72 ± 0.05), PFS (C-index = 0.70 ± 0.04), and PFI (C-index = 0.74 ± 0.06)—significantly higher than clinical-only (OS: 0.66 ± 0.06, PFS: 0.64 ± 0.10, PFI: 0.69 ± 0.06) or ascites-only (OS: 0.69 ± 0.09, PFS: 0.67 ± 0.08, PFI: 0.72 ± 0.09) subsets. This highlights the critical role of integrating multi-dimensional data to capture complex feature interactions influencing survival outcomes.

RSF consistently outperformed DeepSurv, leveraging its tree-based architecture to model non-linear relationships effectively. Single feature sets, particularly ascites-only, showed limited discriminatory power, underscoring their inability to fully characterize disease dynamics alone. These findings emphasize the value of combined features and algorithm selection in robust survival modeling, with RSF offering a superior approach for identifying heterogeneous risk subgroups in translational research.

The observed superiority of combined clinical-ascites features across all survival endpoints underscores the importance of integrative feature selection in capturing multi-dimensional prognostic signals. While single feature groups provided moderate predictive value (e.g., ascites features for OS and clinical features for PFI), their limitations were evident in failing to achieve top performance in any endpoint. This suggests that survival outcomes are influenced by complex interactions between clinical phenotypes and immune microenvironments, which require integrated analysis to fully resolve. Prior work (24, 25) used clinical or imaging data alone and missed immune determinants of survival and therapy resistance. Unlike the NLP model of Laios et al., which relies on operative-report text and cannot reflect pre-operative immune dynamics, our inclusion of markers (e.g., PD-1^+^CD8^+^ T cells) flags immune-exhausted patients prone to R2 residual disease. Compared with the ultrasound model of Moro et al., which is operator-dependent and largely morphologic, our approach links pre-operative immune status to residual tumor diameter, thereby improving survival prediction.

Model comparisons further highlighted the robustness of RSF over parametric CPH and neural network DeepSurv approaches. RSF’s ability to handle non-linear relationships and automatically select informative features contributed to its dominance, particularly in combined feature sets where interactions were most pronounced. In contrast, CPH’s reliance on proportional hazards assumptions and linearity likely constrained its performance, especially in high-dimensional immune features. DeepSurv, despite showing promise in ascites-only models, struggled with overfitting in combined groups, possibly due to insufficient regularization or limited training data. These findings align with recent studies demonstrating ensemble methods’ superiority in survival analysis with heterogeneous data.

For P-DCR, the main message is that fusing information beats any single source. The combined feature set consistently delivered better discrimination (highest mean AUC of 0.69) and a distinct performance profile, suggesting complementary signals between clinical factors and ascites-derived markers. Practically, this favors a combined-features classifier when the goal is to enrich responders while keeping false positives in check. Thresholds should be tuned to the intended use (screening vs. confirmatory), and calibration plus external validation is needed to ensure transportability and avoid optimism from internal resampling. However, similar to the situation in survival prediction, the average sensitivity across all feature sets varied, with the Combined_features group showing the highest mean sensitivity (0.70) while the other groups remained relatively low (0.52–0.54), indicating that even the best-performing model may still miss some resistant samples. This could be due to the heterogeneity of P-DCR features and the model’s limitations in handling complex relationships. In terms of average specificity, the Ascites_features group had a mean specificity of 0.71, outperforming the Combined_features group (0.62) and Clinical_features group (0.69), showing that ascites-derived markers alone have a relatively stronger ability to identify non-resistant samples and reduce false-positive rates.

The SHAP analysis has yielded valuable insights into the significance of diverse features for predicting clinical outcomes in HGSOC. Mechanistically, the presence of PD-1^+^CD57^+^CD8^+^ T cells in ascites indicates terminal differentiation/senescence with features of exhaustion; studies have linked expansion of this lineage to adverse outcomes in HGSOC, consistent with its unfavorable contribution in our models. Moreover, datasets that distinguish PD-1 single-positive CD8 T cells (which may include activated states) from PD-1 and CD57 double-positive cells (more senescent/terminal) show divergent prognostic directions, supporting our interpretation of PD-1^+^CD57^+^ as the more risk-oriented phenotype (29, 30). CCR7^-^CD45RA^+^CD4^+^ (CD4 TEMRA) denotes terminal effector differentiation, generally accompanied by reduced proliferative reserve and immunosenescence; the biological meaning of the CCR7/CD45RA stratification is well established and aligns with the risk direction we observed (31). Tregs (CD25^+^CD127^-^) are recruited to ovarian tumors/ascites via CCL22 and are associated with inferior survival, suggesting an immunologic barrier to chemotherapy response (32). Although CD56^dim^ NK cells are the principal cytotoxic subset, they are often functionally impaired in ascites by soluble factors such as TGF-β1; such dysfunction correlates with poorer outcomes and further supports including NK phenotypes in prediction (33). Among clinical covariates, CA125 reflects tumor burden and is used for relapse monitoring, but initiating chemotherapy solely on the basis of rising CA125 does not improve overall survival, indicating that it functions better as a disease-activity marker than as a stand-alone treatment trigger (34). FIGO stage and residual disease have long been among the most important prognostic factors, in accordance with their directions in our models (35). PARPi exposure was treated as a treatment covariate rather than an outcome; its association with reduced risk is biologically plausible—PARPi can remodel the tumor immune microenvironment, upregulate PD-L1, and activate innate immune pathways—yet confounding must be considered in nonrandomized analyses (30). Disruptions in these cells could impact prognosis, pointing to potential immunomodulatory therapies. PARPi shows a negative influence on OS, PFS, PFI, and P-DCR, implying a protective effect. Understanding its mechanisms could optimize its use and lead to better combination therapies. Biomarkers like “serum CA125” and “Stage” are relevant for PFS. Their positive associations highlight their importance in monitoring and guiding treatment. Regular assessment can help detect progression early.

As part of ongoing research, deep learning has also been explored for predicting post-operative residual tumor status in patients with ovarian cancer. Preliminary findings suggest that deep learning models can be applied effectively to predict the status of residual tumors, with early results indicating an accuracy of 70.83%, a precision of 71.21%, a recall of 70.83%, and an F1 score of 70.89% on the test set. While these findings are still under investigation, they may provide valuable insights into the potential for integrating tumor status prediction into clinical prognosis of tumors, further supporting personalized treatment approaches.

### Limitations and future directions

Despite achieving meaningful results, this study has certain limitations. First, the small dataset size may affect the generalizability of the models, especially for deep learning approaches that require larger datasets for reliable performance. Despite the promising findings in this study, several limitations must be considered. The most significant limitation is the small sample size (*n* = 87), which can affect the generalizability and robustness of the models. A dataset of this size, while adequate for the proof of concept, may not capture the full heterogeneity of patient populations, particularly in HGSOC, where immune microenvironment features may vary across ethnicities and clinical settings. The lack of external validation is a critical concern, as the generalizability of machine learning models is heavily dependent on the diversity and size of the datasets used for testing. We acknowledge that external validation using multi-center datasets is essential to improve the reliability and applicability of the models to broader populations. Furthermore, multi-center validation would address potential biases due to center-specific variations in clinical procedures and pathological assessments.

To mitigate this limitation, we employed a repeated nested cross-validation approach, which helps reduce overfitting and ensures that the feature selection and model training processes are rigorously tested within the available data. This approach, despite the limited sample size, maximized the utility of the data by ensuring that models were trained and evaluated using different subsets, thus improving the stability of the findings.

While external validation is clearly a priority for future work, our use of advanced techniques such as feature screening, multivariable modeling, and SHAP analyses has ensured that the findings are interpretable and robust within the context of this study. These methods allowed us to capture meaningful relationships between clinical features and ascitic immune cell subsets, which will form a valuable foundation for future investigations with larger and more diverse cohorts. Additionally, the data used in this study mainly originated from a single center, lacking diversity in patient populations from different ethnicities or regions, which may limit the applicability of the model. There is also a lack of targeted analysis regarding center-specific variations in surgical procedures and pathological assessment criteria. This introduces potential bias and variability, further limiting the generalizability of the results. Therefore, future studies should explore multi-center validation to address these concerns and improve the external validity of the models. Moreover, a key limitation not previously discussed is the “timeliness of immune indicator detection”. Ascites samples need to be collected during surgery and processed within 1 h to ensure accurate immune profiling. In clinical practice, particularly in primary hospitals, meeting this tight timeframe may not always be feasible, limiting the model’s applicability in these settings. Future studies could explore the impact of refrigerated sample storage or other methods to preserve the samples during transport, allowing for a more flexible detection timeline and broader model application in clinical practice. Future studies could incorporate additional multimodal features, such as genomic or imaging data, to further enhance model prediction performance and generalizability. In particular, with advancements in deep learning and multimodal integration techniques, combining different data types may provide more comprehensive support for the diagnosis and treatment of ovarian cancer. In terms of model interpretability, future efforts could explore other interpretability techniques, such as attention mechanism-based models, to better uncover the complex relationships and interactions among features. This would deepen our understanding of the mechanisms underlying ovarian cancer and provide new insights for developing individualized treatment strategies.

## Conclusion

This study developed a deep survival model and a random forest-based platinum resistance prediction model for ovarian cancer, integrating clinical and immune features. Key findings include the following: (1) The RSF model outperformed deep learning and traditional methods across all survival endpoints; (2) combined clinical-ascites features improved predictive accuracy, validating multi-dimensional data integration; and (3) signature biomarkers were identified for survival and drug resistance. These results provide a novel framework for personalized treatment strategies. Future work includes external validation and multi-omics integration to enhance prognostic precision.

## Data Availability

The datasets presented in this study can be found in online repositories. The names of the repository/repositories and accession number(s) can be found in the article/supplementary material.
