# Supplementary material for: Decoding ascitic immunological niches with multi-modal machine learning reveals prognostic and chemoresistant determinants in ovarian cancer
Source: Front Immunol. 2025 Dec 3;16:1698793. doi: 10.3389/fimmu.2025.1698793 (PMC12708272; doi:10.3389/fimmu.2025.1698793)
Supplement: Supplementary file 1 [file DataSheet1.docx]

Supplementary


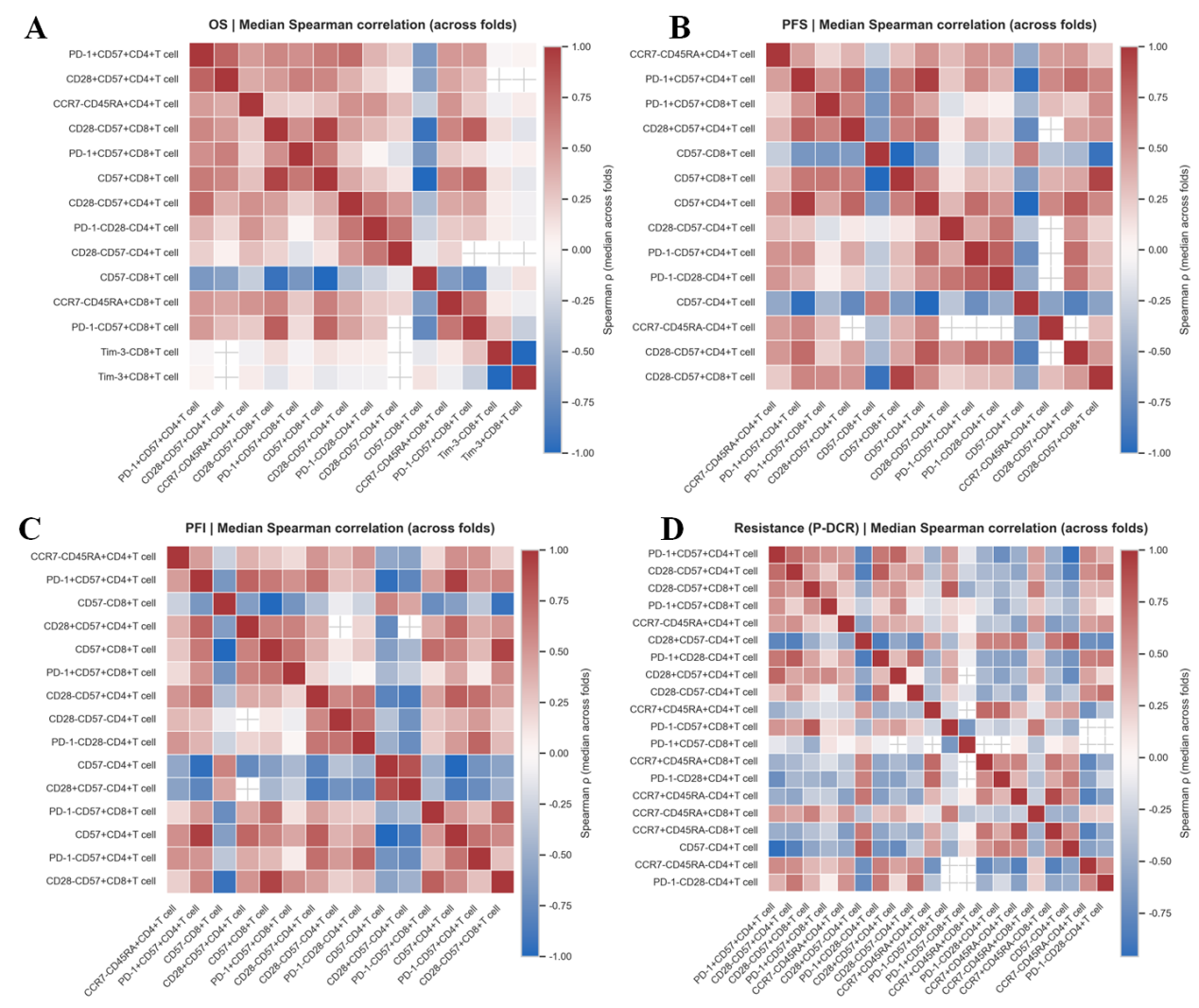


Figure S1. VIF distribution boxplots for immune features associated with each endpoint.


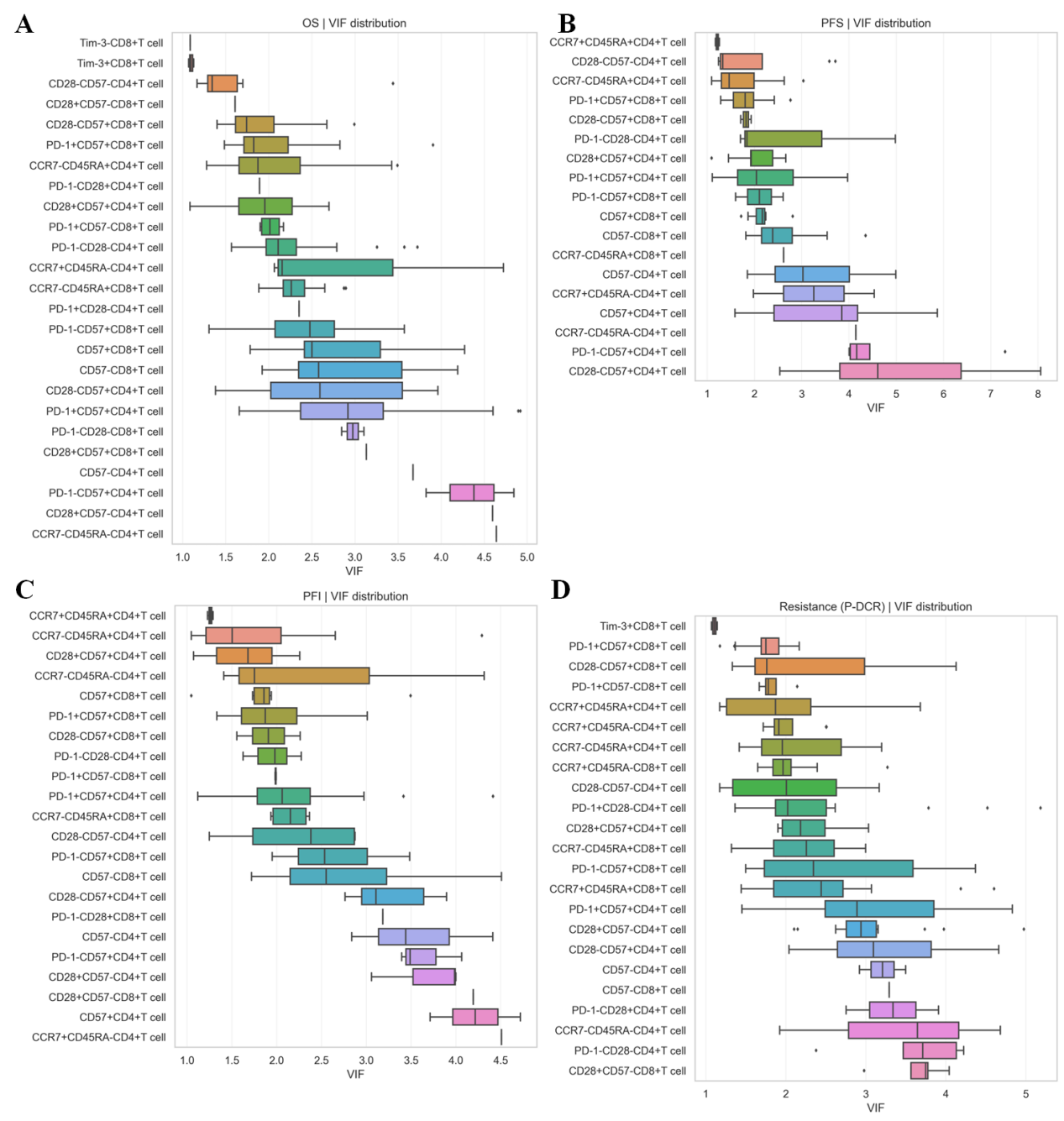


Figure S2. VIF distribution boxplots for immune features across four endpoints (OS, PFS, PFI, P-DCR).


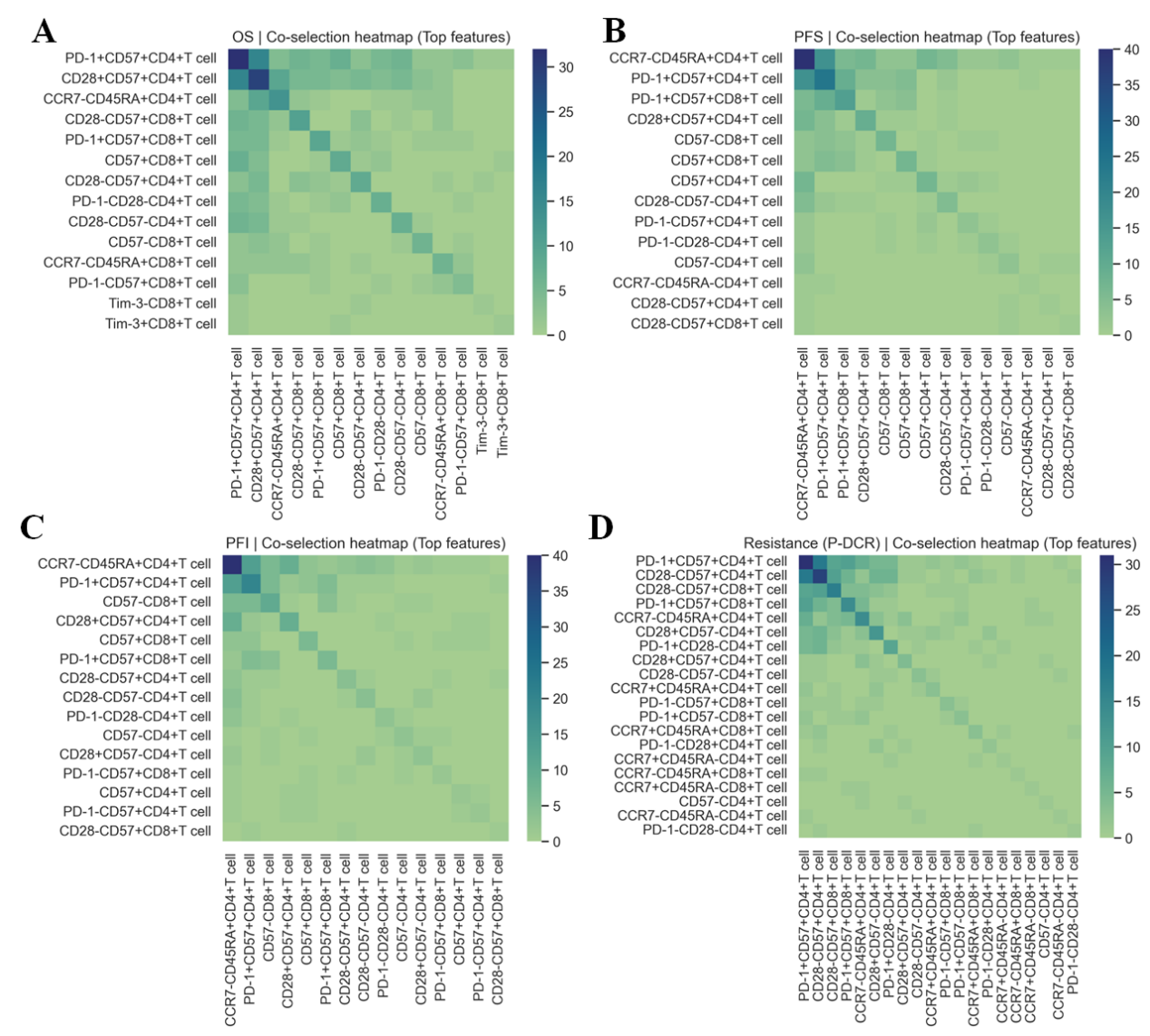


Figure S3. Co-selection heatmaps (A–D). Panels correspond to OS, PFS, PFI, PDCR. Cell (i,j) shows the across-fold count that features i and j were selected together in the same fold; darker color denotes higher co-occurrence.


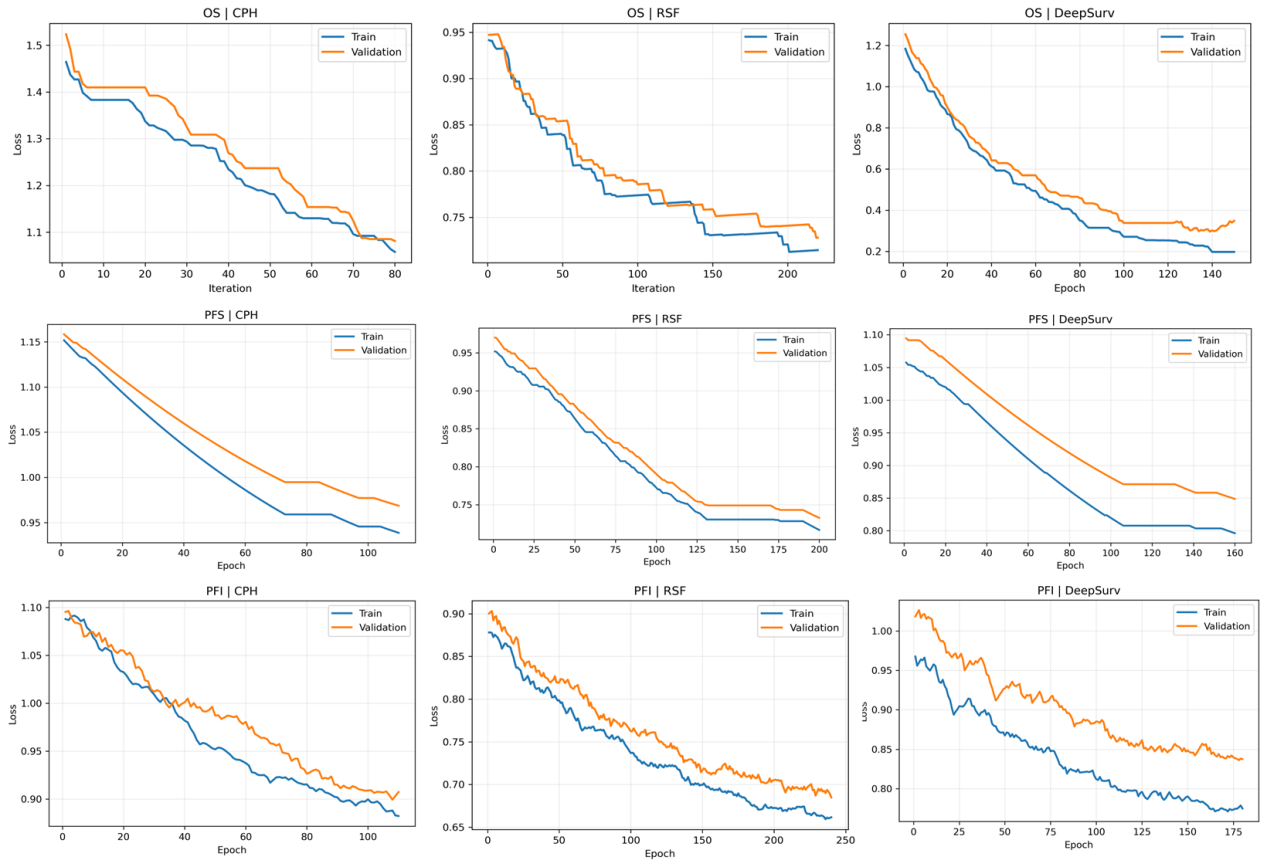


Figure S4. The training and validation loss for OS、PFS、PFI between three models.


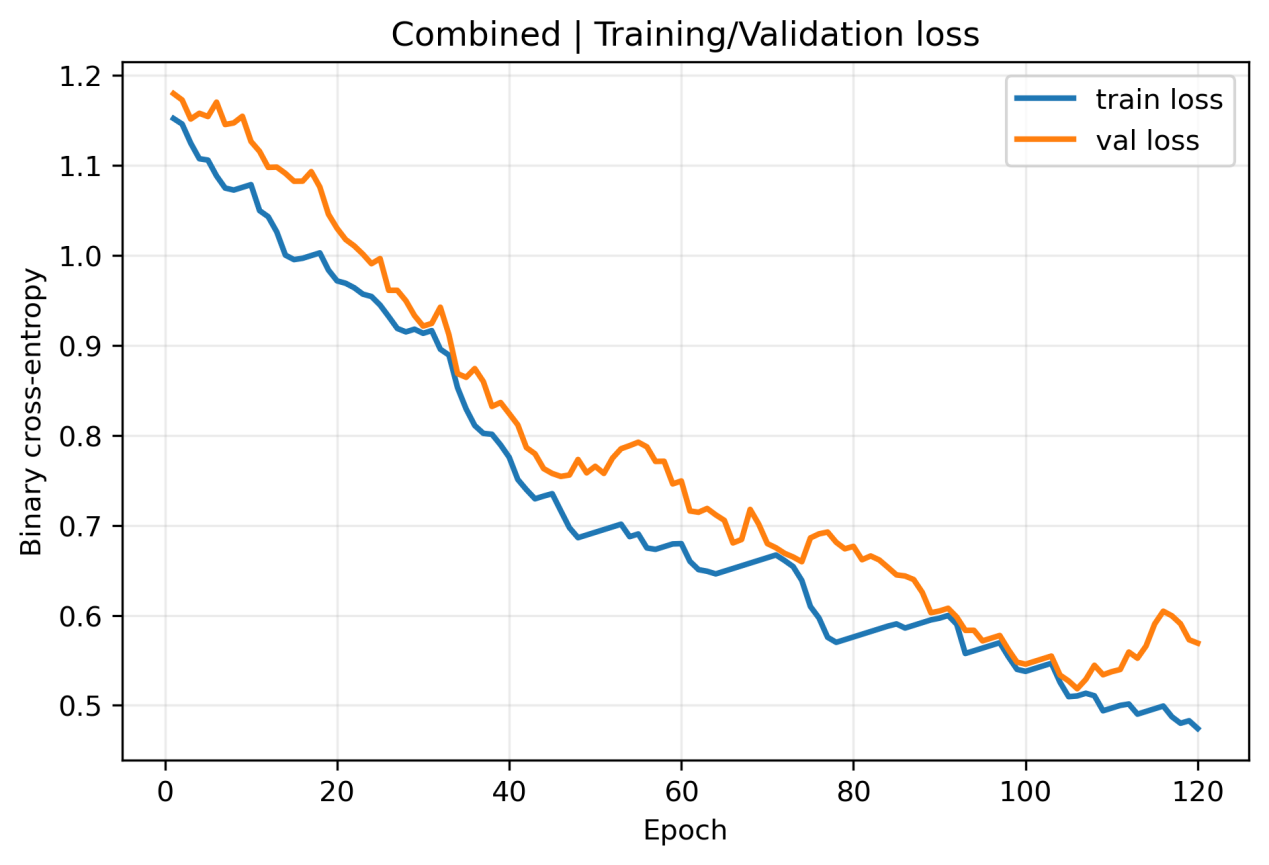


Figure S5. The training and validation loss in P-DCR prediction.
